# Mazu belief and happiness: a sequential mediation model involving mental health and positive emotions among Taiwanese followers

**DOI:** 10.3389/fpsyg.2025.1571796

**Published:** 2025-04-17

**Authors:** Hsiao-Ming Chang, Ching-Hui Lin

**Affiliations:** ^1^Department of Recreational Sport, Putian University, Putian, China; ^2^Office of Physical Education of Yuan Ze University, Taoyuan, Taiwan

**Keywords:** Mazu belief, mental health, positive emotions, happiness, religious culture

## Abstract

**Introduction:**

This study explores the psychological impact of Mazu beliefs on the happiness of Taiwanese followers, emphasizing the sequential mediating roles of mental health and positive emotions. Given the cultural prominence of Mazu belief in Taiwan, this research sheds light on its potential contributions to enhancing well-being from the perspective of positive psychology.

**Methods:**

Using a questionnaire survey, convenience sampling was employed to collect data from followers at 57 Mazu temples across 20 counties and cities in Taiwan. A total of 402 valid responses were obtained. The collected data were analyzed using Partial Least Squares Structural Equation Modeling (PLS-SEM) to evaluate the relationships among Mazu beliefs, mental health, positive emotions, and happiness.

**Results:**

The analysis demonstrated that Mazu beliefs positively and significantly influence mental health and positive emotions. Additionally, Mazu beliefs were found to affect happiness indirectly through the sequential mediation of mental health and positive emotions. These findings support the theoretical framework linking religious belief to emotional and psychological well-being.

**Discussion:**

This study highlights the critical role of culturally embedded religious practices, particularly Mazu beliefs, in fostering mental health and enhancing positive emotions, which collectively promote happiness among followers. These results contribute to advancing the understanding of the intersection between Eastern religious beliefs and positive psychology, offering insights into how spiritual practices can sustain emotional resilience and psychological well-being.

## 1 Introduction

In the era of globalization, academic attention has increasingly turned to the psychological impacts of religious belief on individual well-being and happiness. Religious belief, as a crucial spiritual and emotional resource, has long been linked to improvements in mental health and the cultivation of positive emotions (Abdel-Khalek and Lester, 2018; Van Cappellen et al., 2021). While extensive research has illuminated the positive effects of major institutionalized religions such as Christianity (Dollahite et al., 2018), the emotional and psychological dimensions of Asian folk religions remain underexplored (Aydogdu et al., 2021). This research gap highlights the need to investigate how culturally unique practices of folk religions influence mental health, positive emotions, and happiness.

Among Chinese folk religions, Mazu belief stands out as a historically rich and culturally significant tradition. In 2009, UNESCO designated Mazu belief as an Intangible Cultural Heritage, underscoring its global influence. With over 300 million followers across 46 countries and regions and more than 10,000 registered temples worldwide, Mazu belief has transcended its origins on Meizhou Island, China, to become a central folk religious tradition, particularly in Taiwan (Yao et al., 2020). Originally revered by maritime and fishing communities, Mazu, known as the “Sea Goddess,” was introduced to Taiwan during periods of Chinese immigration and has since evolved into both a spiritual and cultural anchor. Prominent temples such as the Dajia Jenn Lann Temple and Beigang Chaotian Temple serve as hubs of religious activity, hosting vibrant annual pilgrimage festivals that attract millions and reinforce communal bonds among participants (Lee et al., 2021). Beyond its spiritual dimensions, Mazu-related practices integrate social elements, fostering a sense of collective belonging and shared identity within Taiwanese society (Lin, 2021).

Despite the widespread popularity and cultural significance of Mazu belief, its psychological impacts on followers have been insufficiently studied. Unlike Western religious traditions, Mazu belief emphasizes participatory and communal practices, such as oracular divination, temple festivals, and pilgrimages, blending spiritual rituals with social interactions. These practices provide emotional support, strengthen psychological resilience, and cultivate collective identity. For instance, seeking divine advice at Mazu temples is often practiced to gain comfort during distressing times (Zhang, 2017), while temple festivals and religious pilgrimages evoke positive emotions through community engagement and social support (Liu et al., 2023). The unique participatory nature of Mazu belief offers new possibilities for understanding the connections between spirituality, culture, and psychology.

Prior research has established connections between religious belief and well-being (Koburtay et al., 2023), yet the mechanisms underlying these relationships within folk religious traditions like Mazu belief remain unclear (Zhang et al., 2023). Questions persist regarding whether rituals and pilgrimages directly enhance happiness (Liu et al., 2023) or act through mediating factors such as mental health and positive emotions. Additionally, ambiguities in existing literature concerning overlapping terms like “mental health,” “psychological well-being,” and “happiness” underline the need for conceptual and methodological clarification (Lomas et al., 2024). This study addresses these gaps by integrating theoretical analysis with empirical methods to explore how Mazu beliefs—including Belief Practice, Belief Identity, Belief Function, and Belief Idea—impact mental health, positive emotions, and happiness among Taiwanese followers. These findings aim to enrich religious psychology by elucidating the socio-cultural mechanisms underpinning well-being in folk religious contexts and bridging the divide between folk and institutionalized religions.

## 2 Literature review

### 2.1 Theoretical foundation

In recent years, the intersection of psychology and religious belief has garnered increasing academic interest, particularly within the framework of positive psychology (Seligman, 2002). This study explores the psychological implications of Mazu beliefs among Taiwanese followers by drawing on the Broaden-and-Build (B&B) Theory (Fredrickson, 1998) and the Social Support Theory (SST) (Cobb, 1976) as foundational frameworks. Together, these theories offer a nuanced perspective on how religious practice influences mental health, positive emotions, and happiness (Fatima et al., 2018; Fredrickson, 2002; Van Cappellen et al., 2016).

Among Chinese folk religions, Mazu beliefs are a prominent and long-standing tradition. In 2009, it was designated as a UNESCO Intangible Cultural Heritage. With over 300 million followers in 46 countries and regions and more than 10,000 registered temples globally, Mazu beliefs have a far-reaching influence (Yao et al., 2020). Originating from Meizhou Island in China, Mazu, known as the “Sea Goddess” and protector of sailors, was initially popular among fishing and maritime communities. It was introduced to Taiwan during Chinese immigration and has become a central folk religious tradition there (Shuo et al., 2009). Major Mazu temples like the Dajia Jenn Lann Temple and Beigang Chaotian Temple are not only religious institutions but also cultural hubs, hosting annual pilgrimage festivals that attract millions and strengthen community bonds (Lee et al., 2021). Mazu-related practices also play an integrative social role, promoting a sense of belonging and collective identity (Lin, 2021).

#### 2.1.1 Broaden-and-Build Theory

The Broaden-and-Build (B&B) Theory, proposed by Fredrickson (2001), posits that positive emotions “broaden” individuals’ thought – action repertoires, facilitating the development of enduring personal resources that enhance happiness and overall well-being. Within the context of Mazu beliefs, religious rituals such as temple fairs, pilgrimages, and divination practices are hypothesized to evoke positive emotions, including gratitude, hope, and awe. For instance, a follower may experience gratitude through acts of prayer or offerings, or feel awe during the ceremonious pilgrimage journeys, which are rich in cultural symbolism and promote collective joy. These emotional experiences, in turn, act as catalysts for fostering psychological resilience, enhancing interpersonal trust, and developing effective coping mechanisms for navigating life’s challenges (Fredrickson and Joiner, 2002).

Empirical evidence supports the link between religious practices and positive emotional outcomes. For example, the emotional states elicited through rituals often contribute to psychological strengthening (Cohn et al., 2009). For Mazu followers, the transcendent sense of meaning and purpose derived from religious engagement is another notable outcome, aligning with well-documented components of happiness (Kashdan et al., 2008). These experiences are hypothesized to create enduring personal resources that extend beyond momentary emotional states, thereby supporting long-term mental health and happiness.

#### 2.1.2 Social Support Theory

The Social Support Theory (SST) provides a complementary framework, emphasizing the critical role of social bonding in mental health and emotional well-being. According to SST, supportive social networks serve as buffers against stress, enhancing resilience and improving psychological outcomes (Taylor, 2011). Mazu-related practices are inherently communal, with activities such as temple pilgrimages and festival celebrations facilitating extensive social interaction and collective bonding. For example, the renowned Dajia Mazu pilgrimage in Taiwan, which attracts millions of participants annually, demonstrates the robust social support generated through shared religious engagement (Lee et al., 2021; Lin, 2021). Along the pilgrimage route, devotees engage in mutual acts of support, such as organizing food distribution, offering roadside rest areas, and participating in shared worship.

This intricate network of reciprocal aid and shared intention transcends religious devotion, fostering emotional solidarity and collective coping mechanisms. Moreover, it significantly contributes to followers’ sense of life satisfaction, belonging, and psychological refuge (Lin, 2021). For individuals facing personal crises, these practices serve not only as a source of spiritual consolation but also as a profound form of emotional and social refuge, reflecting the applicability of SST in understanding the societal and personal outcomes of Mazu belief systems (Berkman et al., 2000). The communal and interdependent nature of these religious practices highlights their ability to provide unique resources for psychological resilience and happiness.

#### 2.1.3 Integration of theoretical frameworks

This study integrates the Broaden-and-Build Theory (Fredrickson, 2001) and the Social Support Theory (Cobb, 1976) to establish a comprehensive framework for exploring the relationships between religious beliefs, psychological mechanisms, and emotional well-being. The Broaden-and-Build Theory explains how positive emotions elicited through religious rituals, such as temple festivals and pilgrimage activities, enhance individual psychological resilience and happiness by building internal resources (Fredrickson, 2001; Fredrickson and Joiner, 2002; Van Cappellen et al., 2016). Meanwhile, the Social Support Theory highlights the inherently communal nature of Mazu-related practices and how social interactions foster emotional fulfillment and improved mental health (Cobb, 1976; Berkman et al., 2000; Taylor, 2011).

This integrated framework underscores the dual function of Mazu belief: enhancing positive emotions at the individual level while constructing supportive social networks at the communal level. It sheds light on how this Taiwanese folk religion simultaneously fulfills personal and collective psychological needs, providing unique insights into the role of traditional religious practices in fostering emotional well-being. Furthermore, it contributes to the field of religious psychology by situating these findings within the culturally embedded context of Taiwanese folk religion (Yao et al., 2020; Lin, 2021; Fatima et al., 2018).

### 2.2 Religious beliefs and happiness

In psychology, happiness is frequently conceptualized through the framework of subjective well-being (SWB), which comprises both cognitive evaluations (e.g., life satisfaction) and affective experiences (e.g., the presence of positive emotions and the absence of negative emotions) (Diener et al., 2002). Alternatively, subjective happiness is defined as an individual’s immediate self-perception of their happiness state, focusing on their present emotional experience (Lyubomirsky and Lepper, 1999). Both constructs―SWB and subjective happiness―are profoundly influenced by religious engagement, underscoring the critical role of religious beliefs in contributing to overall happiness (Koenig et al., 2012; Saiz et al., 2020).

Research in the field of religious psychology has consistently demonstrated that religious beliefs promote happiness through multiple interconnected pathways. These include fostering a sense of purpose in life, strengthening social relationships, and providing effective mechanisms for emotional regulation (Saiz et al., 2021; Zarzycka et al., 2020). For instance, studies by Abu-Raiya and Ayten (2020) and Park and Kamble (2020) show that religious belief systems, such as those found in Islam and Hinduism, enhance subjective well-being by promoting adaptive coping mechanisms and cultivating social connectedness. These findings form a universal foundation for understanding how religion serves as a multifaceted resource for happiness across diverse spiritual traditions.

In the context of Mazu belief, happiness emerges as a byproduct of its distinctive worship practices and cultural characteristics (Chang A. Y. P. et al., 2020; Chang H. M. et al., 2020). Mazu worship fosters multiple psychological benefits (Chang, A.Y.P. et al., 2020) that align neatly with both SWB and happiness. Specifically, these benefits include:

1. Psychological relief through rituals that address followers’ existential anxieties and uncertainties.
2. A sense of communal belonging, cultivated through collective participation in temple activities, pilgrimages, and large-scale festivals.
3. Expectations of divine intervention, which instill optimism and hope in times of personal or communal need.

Unlike certain religious traditions that impose rigid participation schedules, Mazu followers often engage in rituals in a flexible and need-driven manner, which makes the belief system particularly well-suited to fulfilling the immediate emotional and psychological needs of its adherents. This adaptability aligns closely with happiness, as it emphasizes momentary experiences of satisfaction and relief rather than rigid long-term commitments (Chang A. Y. P. et al., 2020; Chang H. M. et al., 2020). For instance, a follower may choose to seek Mazu’s blessings during periods of uncertainty or personal difficulty, finding instant solace and reassurance through prayers, divination rituals, or communal activities.

Taken together, the theoretical perspectives and empirical findings suggest that Mazu belief is capable of enhancing happiness by providing followers with a unique combination of spiritual, emotional, and social resources. These resources not only promote life satisfaction but also contribute to followers’ day-to-day happiness through immediate and meaningful experiences of joy, relief, and connection. Based on these premises, the following hypothesis is proposed:

*H1*: Mazu beliefs have a positive impact on the happiness of followers.

### 2.3 Religious beliefs and mental health

Religious beliefs, expressed through practices such as prayer, scripture reading, rituals, and other religious behaviors, provide individuals with a framework for interpreting life’s challenges and finding solace during adversity (Hood et al., 2018; Vishkin et al., 2019). These beliefs play a pivotal role in fostering transcendent relationships, creating a sense of belonging, and imbuing life with purpose, all of which are foundational to maintaining mental health (Ben-Nun Bloom et al., 2015; Bosco-Ruggiero, 2020). By offering existential meaning and psychological stability, religion has historically been a significant buffer against the impact of stress and uncertainty.

Empirical studies strongly corroborate the positive relationship between religious beliefs and mental health. For instance, religious engagement has consistently been linked to reduced symptoms of anxiety, depression, and psychological distress, regardless of the religious or cultural context (Koenig et al., 2012; Koenig, 2018). Abdel-Khalek and Lester (2018) noted that religious beliefs enhance resilience and facilitate coping mechanisms, providing individuals with psychological tools to manage life’s adversities. These effects are markedly pronounced in collectivistic cultures, such as Taiwan’s, where religious practices emphasize communal engagement. In such sociocultural contexts, religion often acts as both a spiritual and social support system, reinforcing the psychological well-being of adherents. For example, the rituals and practices associated with Mazu belief, including group participation in temple worship and large-scale pilgrimages, create a shared sense of solidarity and collective psychological reprieve, particularly during times of crisis (Lin et al., 2022).

The unique practices embedded within Mazu belief further amplify its mental health benefits. Centered on the themes of divine protection and guidance, Mazu beliefs encompasses elaborate rituals and grand festivals, such as the Dajia Mazu pilgrimage, which serve as communal spaces for emotional expression and social bonding (Lee et al., 2021). These events not only celebrate cultural identity but also substantially contribute to emotional resilience and social cohesion, as predicted by the Social Support Theory (SST). By fostering collective experiences, these rituals provide psychological relief and reinforce interpersonal connections, helping participants navigate emotional challenges. Martínez de Pisón (2022) observed that religious beliefs can alleviate feelings of guilt and shame, restoring emotional stability. Eastern religious practices, such as those incorporated into Mazu belief, often involve elements of self-transcendence and mindfulness, which have been closely linked to reductions in anxiety and depression, as well as increased life satisfaction (Park and Kamble, 2020).

Taken together, both theoretical perspectives and empirical evidence underscore the potential of Mazu beliefs to support mental health by combining spiritual transcendence with the power of communal bonding. Through its unique rituals and cultural expressions, Mazu beliefs provides followers with psychological tools to cope during adversity, offering both individual and collective pathways to happiness.

Based on these strong theoretical and empirical foundations, this study posits the following hypothesis:

*H2*: Mazu beliefs have a positive impact on the mental health of followers.

### 2.4 Religious beliefs and positive emotions

The relationship between religious beliefs and positive emotions is well-established in contemporary psychology, highlighting the significant role of religion in fostering emotional well-being. Positive emotions—such as gratitude, awe, hope, love, and compassion—constitute the foundation of religious experiences and are critical in reinforcing religious behaviors and practices (Van Cappellen et al., 2021; Braam and Koenig, 2019). Within religious contexts, rituals function as structured mechanisms for eliciting and amplifying these emotional states, thereby contributing to mental and emotional well-being.

The Broaden-and-Build Theory of Positive Emotions (Fredrickson, 1998, 2001) is integrated here to explain how religious beliefs, including Mazuism, influence emotional dynamics. According to this theory, positive emotions “broaden” an individual’s momentary thought-action repertoire, enabling them to generate novel ideas and take adaptive actions. Over time, this broadening effect progressively “builds” enduring personal resources, such as effective coping skills, strengthened social bonds, and heightened resilience (Fredrickson and Joiner, 2002). Within the Mazu belief system, followers frequently experience emotions such as awe during grand processions or pilgrimages, gratitude following successful divination practices, and hope derived from prayer rituals. These emotions serve as mediating mechanisms, deeply intertwined with improved mental health and greater overall satisfaction with life.

Empirical studies further substantiate this relationship between religious beliefs, rituals, and positive emotions. For instance, Vishkin et al. (2019) found that rituals due to their inherent predictability, meaningful structure, and symbolic significance foster emotional stability by mitigating anxiety and enhancing positive emotional states. Religious rituals not only create moments of emotional elevation but also establish an internal sense of peace and security. Similarly, Yih et al. (2020) identified 12 distinct positive emotions commonly elicited by religious rituals, many of which align with the emotional experiences reported by Mazu followers. These findings suggest that the emotional dimensions of religious practices are not incidental but represent central components of their psychological impact.

Given these strong theoretical and empirical foundations, this study hypothesizes the following:

*H3*: Mazu beliefs have a positive impact on the positive emotions of followers.

### 2.5 The relationship between mental health, positive emotions, and happiness

The relationship among mental health, positive emotions, and happiness is introduced within the context of Fredrickson’s Broaden-and-Build Theory. According to this theory (Fredrickson, 1998, 2001), positive emotions serve as a pivotal mechanism that links mental health with happiness in a sequential manner. Positive emotional states such as hope, gratitude, and joy broaden individuals’ cognitive and behavioral repertoires, thereby enhancing psychological resources and fostering resilience (Fredrickson and Branigan, 2005).

In relevant sections, it has been established that religious faith, such as Mazu belief, enhances mental health through its supportive and communal aspects. Building on this foundation, improved mental health can promote the generation of positive emotions. These positive emotions are critical as they alleviate emotional distress, reduce psychological strain, and directly contribute to happiness by increasing life satisfaction and overall emotional well-being (Fredrickson and Joiner, 2002). This emphasizes the role of positive emotions as mediators in the pathway between mental health and happiness, creating a progressive and directional influence, as hypothesized in this study.

While the literature does document significant correlations among mental health, positive emotions, and happiness, it is crucial to emphasize that the present study focuses on these constructs in a unidirectional and sequential framework. Specifically, the hypothesized model posits that better mental health leads to positive emotions (H4), which subsequently foster happiness (H6). Additionally, mental health is hypothesized to have a direct positive impact on happiness as well (H5). This approach aligns with Fredrickson’s (2001) theoretical perspective and avoids framing the relationships as reciprocal interactions or cycles, which would contradict the causal pathway outlined in the structural model.

Religious practices further enrich this framework. The positive emotions generated from Mazu-based practices—such as hope, joy, and gratitude—enhance followers’ psychological resilience and deepen emotional well-being (Chang A. Y. P. et al., 2020; Chang H. M. et al., 2020). In line with this, Van Cappellen et al. (2016) found that religious rituals in general often evoke gratitude, joy, and other positive emotions, which play a significant role in psychological strengthening and emotional fulfillment. For example, communal rituals such as temple pilgrimages and other collective activities foster social connection, emotional support, and gratitude, which are instrumental in promoting mental health and generating positive emotions. As Ellison and George (1994) point out, religious participation often strengthens social ties and promotes emotional support networks, while Durkheim’s (1995) seminal work highlights how collective rituals create a shared sense of belonging and emotional bond among participants. These positive emotions, in turn, serve as mediators that transfer the benefits of mental health into feelings of happiness, further solidifying the sequential relationships proposed in the framework. Fredrickson and Joiner (2002) demonstrated that positive emotions act as mediators between improved mental health and enhanced happiness, aligning with the Broaden-and-Build Theory. Similarly, Lyubomirsky et al. (2005) emphasized that frequent positive emotional experiences significantly boost subjective well-being and overall life satisfaction.

The mediating role of positive emotions is underscored in studies within religious and spiritual contexts. Ramsay et al. (2019) highlight how religious beliefs and practices improve well-being primarily through emotional pathways, while Van Cappellen et al. (2016) provide evidence demonstrating that spiritual practices evoke positive emotions, which regulate the relationship between mental health and life satisfaction. These findings coincide with the experiences of Mazu followers, where the spiritual and communal aspects of Mazu belief amplify positive emotional states, facilitating the pathway from mental health to happiness. This effect is especially pronounced within spiritually driven cultures, such as Taiwan’s, where collective rituals and personal devotion play a significant role in strengthening psychological and emotional resources.

It is important to underscore that hypothesis H4 posits a sequential order where mental health leads to positive emotions before influencing happiness, which is consistent with the overall logic of this study. Improved mental health, fostered through religious faith like Mazu belief, heightens positive emotional experiences, which subsequently contribute to greater happiness. This stepwise causal framework explicitly avoids implying reciprocal interactions or mutually reinforcing cycles but instead emphasizes progressive directionality between constructs.

Based on these theoretical and empirical foundations, this study posits the following hypotheses:

*H4*: The mental health of Mazu followers has a positive impact on positive emotions.

*H5*: The mental health of Mazu followers has a positive impact on happiness.

*H6*: The positive emotions of Mazu followers have a positive impact on happiness.

**Figure 1:**
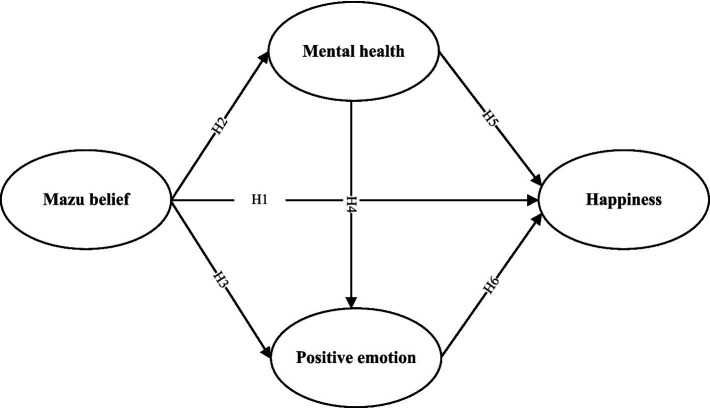
Theoretical model.

Finally, integrating these hypotheses, this study proposes a conceptual framework to examine the stepwise pathways among mental health, positive emotions, and happiness for Mazu followers. The hypothesized relationships are visually represented in Figure 1 to further clarify the proposed causal pathways influencing happiness.

## 3 Research method

### 3.1 Sampling method

This study investigates the followers of Mazu belief and the affiliated temples across Taiwan. Mazu belief, one of the most prominent folk religions in Taiwan, encompasses a vast network of worshippers and temples. Official records indicate that there are more than 500 registered Mazu temples and over 500 unregistered temples nationwide (Platform for Taiwanese Religion and Folk Culture [PTRFC], 2025). According to data from the Taiwan Ministry of the Interior, approximately 60% of Taiwanese—equating to around 13.8 million individuals out of a total population of 23 million—identify as followers or believers in Mazu (PinView, 2022).

This study strictly adheres to the regulations of the academic ethics committee of the author’s university and is carried out with research integrity. To ensure adequate external validity and cultural representativeness, the study selected 57 representative Mazu temples across 20 cities and counties in Taiwan. The selection process was guided by three primary criteria: temple size, regional distribution, and historical influence. This approach ensured comprehensive geographic coverage across the four major regions of Taiwan—northern, central, southern, and eastern—thereby capturing a diverse sample of Mazu worshippers from different social and cultural backgrounds.

A convenience sampling method was employed to recruit participants, with worshippers randomly approached to complete the survey either inside the temples or near their entrances. Although convenience sampling provides flexibility, it is prone to potential sampling biases that may affect generalizability. To minimize these risks and enhance representativeness, the following measures were implemented:

1. Geographical diversity of temple selection: temples were selected to represent the worship practices and cultural characteristics of Mazu followers across different regions of Taiwan.
2. Varied timing of survey distribution: data collection was conducted between January and August 2024, spanning multiple seasons and capturing participants from diverse time periods. For instance, respondents attending annual grand temple festivals were likely to include a wider array of worshippers, thereby improving the representativeness of the sample.
3. Standardized training of research personnel: researchers responsible for questionnaire distribution underwent uniform training to standardize their communication style, tone, and approach. This ensured consistency across research sites and reduced potential interviewer-induced bias.

Following the sampling guidelines proposed by Hair et al. (2019), the minimum required sample size was determined based on the complexity of the research model, including the number of latent variables and paths, as well as the statistical power needed for model testing. Given a model with four latent variables and six paths, Hair et al. suggest collecting 10–20 samples per path, resulting in a minimum sample size of 300. To ensure the robustness of the model and sufficient statistical power, the researchers aimed to gather at least 400 valid responses. Ultimately, a total of 402 completed questionnaires were successfully collected, exceeding the minimum requirements.

During the survey phase, multiple challenges arose, particularly those related to the fieldwork environment. Many temples lacked adequate seating arrangements, as worshippers typically stand during prayer or rituals. Additionally, some participants were hesitant to participate due to ceremonial etiquette, time constraints, or emotional reservations. To address these challenges, the research team adopted a patient and respectful approach, providing clear explanations of the study’s academic significance and encouraging respondents to contribute to the advancement of knowledge regarding the Mazu faith. These efforts culminated in the successful collection of 402 valid responses, ensuring the data’s quality, representativeness, and alignment with the study’s objectives.

### 3.2 Research questionnaire

The research employed a comprehensive questionnaire comprising five sections, each designed to measure key variables and ensure data reliability and validity.

The research questionnaire is divided into five parts. The first part is the Mazu Belief Scale, which consists of four factors: belief practice, belief identity, belief function, and belief idea, comprising a total of 16 items. Belief practice refers to behaviors such as participating in Mazu temple fairs, activities, prayers, and other related rituals. Belief identity involves the recognition of and emotional connection to Mazu’s spirit and teachings. Belief function pertains to the psychological support and life guidance derived from Mazu beliefs. Finally, belief idea is concerned with the understanding and identification with doctrinal and cultural values. The design of this scale primarily draws on the Internal Religious Motivation Scale (IRM) developed by Hoge (1972), complemented by pertinent studies (e.g., Leite et al., 2023; Theodori and Robinson, 2019) and extensive firsthand experience with Mazu belief practices. This ensures the scale is well-tailored to reflect the cultural context and lived experiences of Taiwanese believers.

The development of this scale fully incorporates the cultural, historical, and ritualistic traditions central to the worship of Mazu by Taiwanese followers. It has also been adapted and translated for use in religious faith surveys in Taiwan, as demonstrated in several related theses focusing on local religious studies, where it has shown strong reliability, validity, and a robust factor structure. However, in applying this scale specifically to the study of Mazu belief, additional measures were taken to ensure its relevance and applicability within this cultural framework. Several rounds of focus group discussions and expert consultations were conducted with Taiwanese religious scholars, followers of Mazu, and temple personnel. These collaborative efforts ensured the scale not only reflects the unique beliefs and practices of Mazu followers but also contributes to a broader understanding of religious belief systems within an Eastern cultural context.

The second part of the questionnaire was the Mental Health Scale, which evaluates both positive and negative mental states. This scale, consisting of eight items, was adopted from the widely-used Taiwan Social Change Survey (TSCS), developed and administered by Academia Sinica (2020). The TSCS is a long-term, multi-wave research initiative focused on tracking social changes in Taiwan, encompassing various facets of psychological well-being. The specific items in this mental health scale measure essential dimensions such as sleep quality, life burden, self-confidence, hope for the future, emotional tension, family relationships, and general optimism. These items broadly correspond with established theoretical frameworks of mental health, particularly those emphasizing positive psychological states—such as optimism and self-confidence—as well as the alleviation of negative states, like stress and life burden. The Mental Health Scale utilized in this study forms part of the TSCS project and has undergone rigorous testing in various large-scale surveys of the Taiwanese population. As a government-funded and academically supported initiative, the TSCS has consistently validated its instruments for reliability and cultural relevance.

The third section of the questionnaire employed the Positive Emotion Scale, which was designed to measure two distinct dimensions: inner emotional enrichment and positive coping and gratitude mentality, comprising a total of 12 items. The dimension of inner emotional enrichment includes emotions such as joy, hope, and love, while the dimension of positive coping and gratitude mentality encompasses gratitude, compassion, and calmness. This scale was adapted from Van Cappellen et al. (2016), and its development is theoretically grounded in foundational frameworks by Fredrickson (2013), Yih et al. (2020), as well as Seligman (2011) and Lazarus (1991). These theoretical perspectives provide strong support for the scale’s holistic approach to capturing the essence of positive emotions and their influence on mental health and happiness.

The fourth section utilized the Happiness Scale, which primarily assessed Mazu followers’ happiness. This scale consisted of three items and was adapted from the work of Lyubomirsky and Lepper (1999), as revised by Krause et al. (2018). The items required Mazu followers to self-evaluate their happiness levels, compare those levels to their peers, and reflect on their ability to derive the maximum value from life experiences.

All items in the questionnaire were measured on a five-point Likert scale with response options ranging from “strongly disagree” (1) to “strongly agree” (5). This standardized measurement approach ensured methodological consistency and comparability across all sections of the instrument, facilitating a robust analytical framework for exploring the relationships between Mazu belief and key psychological constructs such as mental health, positive emotions, and happiness.

The final section covered demographic variables, collecting data on participants’ gender, marital status, age, educational level, personal monthly income, and occupation. These variables were included to provide a comprehensive profile of the respondents and allow for subgroup analyses, facilitating a deeper understanding of variability within the sample.

### 3.3 Data analysis

This study used SPSS 21.0 to analyze the demographic variables of the Mazu followers. In addition, this study used the partial least square method (PLS-SEM) of Warp PLS 8.0 to test the reliability and validity of Mazu beliefs, mental health, the positive emotion and happiness scales, and the relationships among the four variables. Each indicator was evaluated according to the PLS-SEM test standard in Kock’s (2023a,b) Warp PLS 8.0 manual. Reliability was tested by component reliability (CR) and Cronbach’s alpha, both of which needed to reach or exceed 0.70. The validity evaluation was based on a factor loading of 0.50 and an average variance extraction (AVE) of or above 0.50 to ensure convergent validity. The differential validity AVE square root needed to be greater than or equal to 0.70. In the model structure relationship, the significance of path coefficients was analyzed and the explanatory power of the model was evaluated using *R*^2^ (models where the *R*-squared coefficients or adjusted *R*-squared coefficients were below 0.02). These rigorous statistical analyses were used to ensure the reliability and validity of the research results.

### 3.4 Confirmatory factor analysis

According to the analysis results presented in Table 1, for the four dimensions of Mazu Beliefs, the factor loadings of all items exceeded 0.70, indicating satisfactory measurement properties. These four latent dimensions—Belief Practice, Belief Identity, Belief Function, and Belief Idea—refer to distinct conceptual variables specifically aligned with the psychological and behavioral aspects of Mazu worship. The alignment between latent dimensions and their corresponding conceptual variables is outlined below:

1. Belief Practice, representing Religious Activities focuses on the external behaviors followers engage in, such as temple attendance, ritual participation, and prayer practices. This dimension emphasizes the performative and communal aspects of Mazu faith.
2. Belief Identity, reflecting Personal Identification with Mazu Beliefs, highlights the psychological attachment and internalization of Mazu teachings, values, and shared beliefs between followers.
3. Belief Function corresponds to Transformative Religious Outcomes, capturing followers’ perceptions of Mazu beliefs as regulating their behavior, fostering altruism, forgiveness, and responsibility.
4. Belief Idea, representing Spiritual Guidance and Inspiration, measures the ideological and symbolic support derived from Mazu worship, including providing strength, perseverance, and existential direction.

**Table 1:** Summary of confirmatory factor analysis results for the scale.

Latent variables	Observed variables	Factor loading	CR	Cronbach^’^s alpha	AVE
Belief practice	1. I often go to the temple to worship Mazu and pray for safety and smoothness.	0.85	0.89	0.84	0.68
	2. I participate in various activities at the Mazu Temple, such as pilgrimages, ritual reform, religious ceremonies, and volunteering.	0.64			
	3. When encountering setbacks and difficulties, I will go to the temple to pray for Mazu’s help.	0.90			
	4. When making major decisions, I will go to the temple to seek advice from Mazu.	0.89			
Belief identity	5. The belief in Mazu has a great impact on my daily life.	0.81	0.91	0.86	0.71
	6. I agree with the teachings of all Mazu beliefs.	0.83			
	7. I have a strong belief in Mazu	0.87			
	8. I share my Mazu beliefs with others.	0.85			
Belief function	9. Mazu beliefs regulate my behavior.	0.84	0.94	0.91	0.80
	10. Mazu beliefs make me more responsible.	0.92			
	11. Mazu beliefs make me willing to forgive others.	0.89			
	12. Mazu beliefs make me more willing to help others.	0.90			
Belief idea	13. Mazu beliefs guide me to do more good deeds.	0.86	0.94	0.91	0.79
	14. Mazu beliefs guide my life direction.	0.89			
	15. Mazu beliefs provide me with spiritual support.	0.91			
	16. Mazu beliefs give me the strength to persevere.	0.90			
Mental health	1. I feel like I cannot sleep well.	0.88	0.96	0.95	0.79
	2. I think many things are a burden for me.	0.89			
	3. I feel like I have lost confidence in myself.	0.92			
	4. I feel that life is hopeless.	0.89			
	5. I feel nervous and restless, unable to relax.	0.93			
	6. I think my family or friends will make me worry.	0.82			
	7. I think I get along well with my family and friends.	0.30*			
	8. I feel that the future is full of hope.	0.47*			
Inner emotional enrichment	1. I feel happy.	0.42*	0.93	0.91	0.73
	2. I feel there is hope in life.	0.84			
	3. I have emotions in my heart.	0.90			
	4. My heart is full of love.	0.89			
	5. I feel pleasure.	0.83			
	6. I am in awe.	0.79			
Positive coping and gratitude mentality	7. I have a grateful heart.	0.82	0.93	0.91	0.72
	8. My life is more challenging.	0.85			
	9. I have empathy.	0.88			
	10. I feel relieved.	0.84			
	11. I find peace.	0.37*			
	12. I am more determined to do things.	0.87			
Happiness	13. Compared to most people of the same age group, I believe I am happier.	0.88	0.83	0.68	0.62
	14. I am a very happy person.	0.57			
	15. Anyway, I like my current life.	0.88			

*The factor loading for this item did not reach 0.05; therefore, it was deleted.

These mappings ensure that the latent variables measured in Table 1 are accurately connected to their underlying theoretical constructs, thereby validating the multi-dimensional structure of the Mazu Belief Scale as well as its direct implications for mental health, positive emotions, and happiness. Among these dimensions:

1. Belief Practice demonstrated acceptable reliability and validity, with a Composite Reliability (CR) of 0.89, a Cronbach’s alpha (*α*) of 0.84, and an Average Variance Extracted (AVE) of 0.68.
2. Belief Identity exhibited similarly strong reliability and validity, with CR = 0.91, Cronbach’s *α* = 0.86, and AVE = 0.71.
3. Belief Function achieved excellent reliability and validity, with CR = 0.94, Cronbach’s *α* = 0.91, and AVE = 0.80.
4. Belief Idea also demonstrated high reliability and validity, with CR = 0.94, Cronbach’s *α* = 0.91, and AVE = 0.79.

Further analysis of the Mazu Belief Overall Scale revealed strong reliability and good convergent validity, with composite reliability (CR) = 0.94, Cronbach’s alpha = 0.94, and average variance extracted (AVE) = 0.84. These results indicate that the scale is both robust and effective in capturing the intended dimensions of Mazu belief.

Regarding the mental health scale, the analysis revealed that the factor loadings for Item 7 and Item 8 did not exceed the threshold of 0.5. These two items were positively worded, while the rest of the scale consisted of reverse-coded items. We believe that the primary reason for their low factor loadings may be their conceptual similarity to the meaning of the first six reverse-coded items. Additionally, although Item 8 exhibited a factor loading of 0.47 (*p* < 0.05), it was excluded from the scale due to its conceptual overlap with Item 2 from the internal emotional enrichment factor under the positive emotions domain. After removing these two items, the recalibrated six even-item scale demonstrated improved reliability and validity compared to the original eight-item version, with composite reliability (CR) = 0.96, Cronbach’s *α* = 0.95, and average variance extracted (AVE) = 0.79, indicating strong reliability and good convergent validity.

On the positive emotion scale, the analysis revealed that items 1 and 11 had factor loadings below the threshold of 0.50 and were consequently removed from the construct. After the removal of these two items, the re-estimated “internal emotional enrichment” construct displayed improved reliability and validity compared to the original six-item model, with composite reliability (CR) = 0.93, Cronbach’s alpha = 0.91, and average variance extracted (AVE) = 0.73. Moreover, the constructs of positive coping and gratitude mentality also demonstrated enhanced psychometric properties relative to the original six-item model, with CR = 0.93, Cronbach’s alpha = 0.91, and AVE = 0.72. Further analysis of the overall positive emotion scale revealed strong reliability and convergent validity for the entire construct, with CR = 0.94, Cronbach’s alpha = 0.86, and AVE = 0.88. These results indicate that the positive emotion scale is robust and effective in measuring its intended dimensions.

The Composite Reliability (CR) of the happiness scale was calculated as 0.83, and the Cronbach’s α value was 0.68, which is slightly below the conventional threshold of 0.70 but remains close to the acceptable range. Despite the marginal deviation in Cronbach’s α, the scale exhibits sufficient reliability when viewed alongside other indicators. In terms of validity, analysis results revealed that the factor loadings for each observed variable exceeded 0.50, with the Average Variance Extracted (AVE) reaching 0.62. These values indicate strong convergent validity, demonstrating that the items within the scale effectively measure the underlying construct. Furthermore, the consistent factor loadings (all >0.50) and AVE (>0.50) reinforce the convergent validity of the happiness scale, substantiating its suitability for use within this study. When reliability and validity indicators are combined—specifically a CR of 0.83 and robust factor loadings—the findings confirm that the happiness scale is a reliable and valid instrument for assessing individuals’ feelings of happiness among Mazu followers.

## 4 Results

### 4.1 The socio-demographic profile of the respondents

Of the 402 participants with valid questionnaires, 198 (49.3%) were male and 204 (50.7%) were female. In terms of marital status, 220 people were married (54.7%) and 182 were unmarried (45.3%). In terms of age distribution, the largest age group was 51-60 (82 people, 20.4%), followed by the age group 21-30 (82 people, 20.4%), while the smallest age group was below 20 (32 people, 8%). In terms of education level, the number of people with a university degree was the highest (165 people, 41%), while the number of people with a graduate degree was the lowest (41 people, 10.2%). In terms of occupation, business employees accounted for the largest number (80 people, 19.9%), while medical staff had the smallest number, (six people, 1.5%). In terms of personal monthly income (in New Taiwan Dollars, NTD), the number of people with an income between NTD 30,001 and NTD 50,000 was the highest (160 people, 39.8%), while the number of people with an income between NTD 80001-100000 was the lowest (seven people, 1.7%) (Table 2).

**Table 2:** Respondent profile.

Demographic characteristics	Frequency	Percentage (%)
Gender		
Male	198	49.3
Female	204	50.7
Marital status		
Married	220	54.7
Unmarried	182	45.3
Age		
Under 20	32	8
21–30	82	20.4
31–40	80	19.9
41–50	79	19.7
51–60	82	20.4
61 and over	47	11.7
Education level		
Primary	64	15.9
High school	89	22.1
Junior college	43	10.7
University	165	41
Postgraduate	41	10.2
Occupation		
Military, governmental employees and teachers	69	17.2
Industrial and commercial service industry	80	19.9
Manufacturing industry	49	12.2
Housekeepers (the retired)	67	16.7
Medical staff	6	1.5
Students	63	15.7
Free trade and transportation Industry	40	10
Agriculture, forestry, fishing, and animal husbandry	28	6.3
Monthly income (NT$)^a^		
Non-income	79	19.7
≦20,000	73	18.2
20,001–40,000	160	39.8
40,001–60,000	63	15.7
60,001–80,000	9	2.2
80,001–100,000	7	1.7
≧100,001	11	19.7

^a^USD$1 = NTD$30.

### 4.2 Discriminant validity analysis

From Table 3, it can be seen that the AVE square root of all potential variables in the study model was greater than 0.70 (between 0.79 and 0.92) and the AVE of each potential variable was greater than all correlation coefficient values in the same column, which met the testing criteria and indicated that the measurement model in this study had good discriminative validity.

**Table 3:** Discriminant validity analysis.

Latent variables	Mazu beliefs	Mental health	Positive emotion	Happiness
Mazu belief	**0.92**	0.19	0.71	0.51
Mental health	0.19	**0.83**	0.30	0.37
Positive emotion	0.71	0.30	**0.94**	0.68
Happiness	0.51	0.37	0.68	**0.79**

The value of diagonal line is the square root of the average variance extraction of each dimension.

### 4.3 Model fit and robustness tests

To evaluate the suitability of the structural equation model (SEM) for further analysis, the overall model fit and robustness were assessed using WarpPLS 8.0 software. The key fit indices are detailed as follows:

(1) *R*-squared contribution ratio (RSCR):

The RSCR for the model was 1.000, exceeding the recommended threshold of ≥ 0.9 and achieving the ideal value of 1. This indicates that the model does not display bias in the explanatory power of the endogenous variables.

(2) Average full collinearity VIF (AFVIF):

The AFVIF was 1.973, which is well below the maximum acceptable threshold of 5 and falls within the ideal range of ≤ 3.3. This suggests that multicollinearity is not an issue among the variables in the model. Furthermore, the Full Collinearity Variance Inflation Factors (VIFs) test was conducted to assess common method bias, as recommended by Kock (2023b). Compared to traditional methods relying on exploratory and confirmatory factor analysis, this test is considered more conservative and effective in detecting and mitigating potential issues of common method bias (Kock, 2023a). The analysis revealed specific VIF values for key constructs: Mazu belief = 2.004, mental health = 1.16, positive emotion = 2.76, and happiness = 1.97. All values were below the recommended threshold of 3.3, confirming compliance with the accepted standards. This indicates that common method bias is unlikely to distort the validity of the structural model or inflate relationships among the constructs.

(3) Tenenhaus GoF (GoF):

The goodness-of-fit (GoF) value was 0.52, surpassing the recommended threshold (≥0.36) for a “large” model fit. This result confirms the strong overall goodness-of-fit for the model.

(4) Average path coefficient (APC):

The APC was 0.313, with statistical significance at *p* < 0.001, indicating that the relationships among the latent variables in the model are significant.

These diagnostic results collectively confirm that the sample size utilized in this study is sufficient to meet theoretical and empirical requirements. Furthermore, the structural equation model displays strong statistical robustness and adaptability, supporting its appropriateness for hypothesis testing in this context.

### 4.4 Structural model analysis

**Figure 2:**
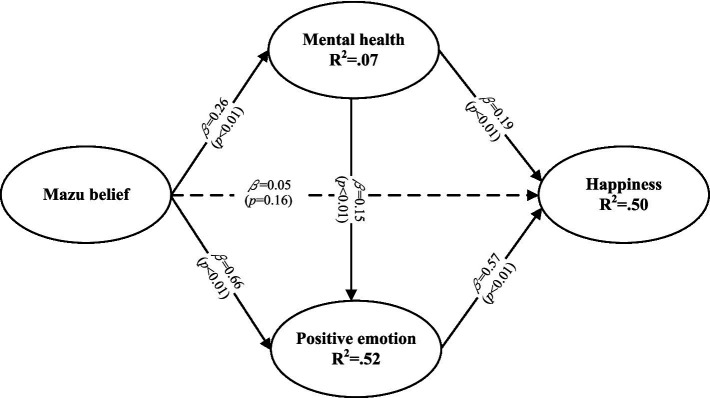
Structural model.

The path coefficients derived from the structural model (standardized regression coefficients, *β* values) are presented in Figure 2. The key findings are summarized as follows:

1. Mazu beliefs had a significant, positive impact on mental health (*β* = 0.26, *p* < 0.01) and positive emotions (*β* = 0.66, *p* < 0.01).
2. Mental health showed a significant, positive impact on both positive emotions (*β* = 0.15, *p* < 0.01) and happiness (*β* = 0.19, *p* < 0.01).
3. Positive emotions exerted a strong, positive influence on happiness (*β* = 0.57, *p* < 0.01).
4. However, no direct relationship was found between Mazu beliefs and happiness (*β* = 0.05, *p* = 0.16), as the path was not statistically significant.

These results indicate that the influence of Mazu beliefs on happiness is mediated through mental health and positive emotions rather than having a direct effect. This suggests that the psychological benefits of Mazu belief operate primarily through its contributions to improved mental well-being and enhanced positive emotional experiences among followers, which in turn contribute to their sense of happiness.

The *R*^2^ value represents the predictive power of a research model. In this study, Mazu beliefs had a predictive power of 7% for mental health and 52% for positive emotion. The predictive power of mental health and positive emotion for happiness reached 50%.

WarpPLS calculated three meaningful fitting indices under variance-based SEM: the average path coefficient (APC), the average *R*-squared (ARS), the average adjusted *R*-squared (AARS), and the average block variance inflation factor (AVIF). The values for APC and ARS should both be below 2 and have statistical significance (*p* < 0.05), while the value for AVIF is recommended to be below 5 (Kock, 2023a). The entire model of this study showed an acceptable fit, with APC = 0.31 (*p* < 0.001), ARS = 0.36 (*p* < 0.001), AARS = 0.36 (*p* < 0.001), and AVIF = 1.51. Table 4 reports the results of the hypothesis testing.

**Table 4:** The results of the hypothesis tests.

Hypothesis	Path coefficient (*β*)	Test result
H1: Mazu beliefs → Happiness	0.05	No Support
H2: Mazu beliefs → Mental health	0.26**	Support
H3: Mazu beliefs → Positive emotion	0.66**	Support
H4: Mental health → Positive emotion	0.15**	Support
H5: Mental health → Happiness	0.19**	Support
H6: Positive emotion → Happiness	0.57**	Support

Model fit indicators: APC = 0.31 (*p* < 0.001), ARS = 0.36 (*p* < 0.001), AARS = 0.36 (*p* < 0.001), AVIF = 1.51 (acceptable if <= 5, ideally <= 3.3). **P < 0.01.

The goodness-of-fit results presented above reveal a robust interconnection between Mazu belief, mental health, positive emotion, and happiness. The model fit indices confirm that the structural equation model possesses strong explanatory power, substantiating the relationships among these constructs.

Although Mazu belief does not exert a direct influence on happiness, it has been found to have a significant direct impact on both mental health and positive emotions. Additionally, both mental health and positive emotions demonstrate significant direct effects on happiness. According to Kock (2023a), examining indirect and total effects provides key insights into how mediating variables contribute to the relationships among constructs. Upon analysis, it was identified that while Mazu belief does not directly influence happiness (*β* = 0.05), it indirectly affects happiness through mental health (*β* = 0.05). Furthermore, Mazu belief also indirectly impacts happiness through positive emotions (*β* = 0.38). When considering both direct and indirect pathways, the total effect of Mazu belief on happiness is substantial (*β* = 0.48).

These findings highlight the psychological mechanisms underlying the influence of Mazu belief. Specifically, while Mazu belief does not exert a direct impact on happiness, it significantly enhances happiness through its pronounced effects on mental health and positive emotions, which serve as critical mediating variables.

## 5 Discussion

This study demonstrates that Mazu belief significantly enhances followers’ mental health, a finding consistent with previous research highlighting the positive psychological impacts of religious faiths. Similar to studies on other major religions, such as Christianity, Islam, and Hinduism (Koenig, 2018; Park and Kamble, 2020; Martínez de Pisón, 2022), the results suggest that Mazu followers can alleviate emotional burdens and strengthen mental health by engaging with their belief system. Key processes include fostering hope for the future, managing life pressures, reducing anxiety surrounding familial obligations, and fostering emotional stability. Supporting these findings are religious coping theories (Dolcos et al., 2021; Triana and Sudjatmiko, 2021), which emphasize that faith is often a crucial psychological and spiritual resource, helping individuals navigate life’s challenges effectively.

The study further identifies a positive association between mental health and followers’ positive emotions. This aligns with broader literature on the connection between psychological well-being and emotional states (Fredrickson, 2002; Park and Kamble, 2020). Enhanced mental health fosters states of enthusiasm, optimism, and gratitude, which, in turn, cultivate richer positive emotional experiences. This observation is consistent with Fredrickson’s Broaden-and-Build Theory (B&B Theory) (1998), which posits that positive emotions broaden one’s cognitive and behavioral repertoire, initiating upward spirals of well-being and resilience.

Although this study utilizes a single-factor framework for parsimony and simplicity, future research could benefit from exploring the distinct dimensions of these constructs. For instance, internal emotional enrichment, encompassing states such as joy, contentment, and spiritual peace, may exhibit a direct relationship with mental health. On the other hand, adaptive coping strategies, such as gratitude and cognitive reframing, might mediate the pathway between mental health and happiness (Aldwin et al., 2014; Kidwai et al., 2014). These distinctions could illuminate how specific belief practices—such as temple worship, prayer to Mazu, and participation in cultural festivals—enhance psychological well-being while fostering emotional adaptability.

The findings confirm that Mazu belief does not exert a direct effect on happiness; rather, its effects are mediated by mental health and positive emotions. This aligns with recent studies indicating that religious belief often affects subjective well-being through mental health improvements and emotional regulation (Koenig, 2018; Diener et al., 2002; Lyubomirsky and Lepper, 1999). To understand why this is the case, it is essential to consider the nature of Mazu belief and the psychological processes involved. Mazu belief, deeply rooted in Taiwanese culture, serves as a spiritual anchor and source of comfort for its followers, similar to other forms of religious coping observed across cultures (Lin, 2021). Such beliefs provide followers with a framework for understanding the world and coping with life’s uncertainties, helping them to mitigate anxiety and stabilize their emotional states. This cultural and spiritual dimension uniquely positions Mazu belief as not only an individual psychological resource but also a collective framework for enhancing social and emotional well-being (Yao et al., 2020).

Happiness, however, is a complex and multi-faceted construct that encompasses both emotional and cognitive elements (Diener et al., 2018). Spiritual belief alone cannot fully define happiness, as it involves an overall subjective evaluation of one’s life satisfaction and well-being, influenced by emotional resilience and positive mental states (Koenig, 2018; Saiz et al., 2021). Mazu belief primarily operates on the psychological and emotional levels, influencing followers’ mental states by providing hope, reducing anxiety, and fostering a sense of security (Chang A. Y. P. et al., 2020; Chang H. M. et al., 2020).

Positive emotions are a crucial by-product of Mazu belief. Empirical studies have demonstrated that participation in religious or cultural rituals often leads to enhanced joy, enthusiasm, and gratitude, which are critical for emotional flourishing (Fredrickson, 2001; Van Cappellen et al., 2016). Practices such as temple worship and Mazu-related cultural festivals create a sense of community and belonging among followers, a social dimension strongly associated with improved life satisfaction and stress management (Koenig, 2018; Lin, 2021). These socio-cultural activities facilitate positive affect and community bonding, which indirectly enhance mental health and pave the pathway from Mazu belief to happiness.

This indirect effect is consistent with the broader understanding of how religious beliefs influence well-being. Previous research on other religious traditions has also indicated that religious practices enhance subjective well-being indirectly by building psychological and emotional resilience (Fatima et al., 2018; Ramsay et al., 2019). For instance, in some religious communities, regular religious rituals and social support systems help members develop better coping mechanisms, which then contribute to their overall happiness.

These mediating effects provide further support for Fredrickson’s Broaden-and-Build Theory, highlighting how positive emotions triggered by spiritual and religious practices expand psychological resources and promote resilience (Fredrickson, 2001; Fredrickson and Joiner, 2002). Additionally, they align with Self-Determination Theory (SDT) (Deci and Ryan, 1980), wherein Mazu belief fulfills intrinsic psychological needs such as emotional security, autonomy, and relatedness, thereby contributing indirectly to happiness. These findings emphasize the central role of religious faith in fostering adaptive psychological processes, particularly within unique socio-cultural contexts.

Studies on Buddhism, Taoism, and other Chinese folk religions consistently demonstrate that religious belief functions as a spiritual and emotional anchor, contributing significantly to mental health and happiness (Hou et al., 2023). For instance, research on Daoist practices in Taiwan has shown that rituals, ancestor worship, and deity veneration improve psychological resilience and life satisfaction (Chen et al., 2023). Furthermore, within collectivistic cultural contexts, religious practices often enhance familial and social connections, which foster positive emotions and overall well-being (Saiz et al., 2021).

Comparative studies also provide critical insights. A study on Chinese Buddhism in Hong Kong (Tsui et al., 2020) has revealed that meditation and doctrinal teachings facilitate emotional regulation and cognitive reframing, findings that parallel the effects of Mazu-related practices identified in this study. Similarly, research on Shinto rituals in Japan (Shimazono, 2011) demonstrates how religious practices bolster mental resilience, offering compelling parallels to the coping mechanisms strengthened through Mazu worship.

One novel aspect worth exploring in future studies is the unique feminine and protective archetype of Mazu. Unlike patriarchal deities in Chinese Buddhism or hierarchical practices in Hinduism, Mazu’s maternal symbolism may provide distinctive emotional and psychological benefits. Her representation as a nurturing and protective figure likely resonates deeply with Taiwanese followers, offering a sense of intimacy and communal solidarity. This nuanced cultural and spiritual dynamic highlights potential differences in how religious beliefs operate across various traditions.

From a theoretical perspective, this study contributes to the integration of Eastern religious traditions into Western psychological frameworks. By extending Fredrickson’s Broaden-and-Build Theory and Koenig’s religious coping model (Koenig et al., 2012) to the context of collectivistic cultures, the study underscores the relevance of these frameworks in understanding well-being among Taiwanese and other Eastern populations. Additionally, cross-cultural research on subjective well-being and religiosity (Diener, 1984; Hou et al., 2023) emphasizes the importance of culturally congruent research while revealing unique socio-cultural mechanisms through which happiness is constructed in Eastern contexts.

By bridging psychological universality and cultural specificity, this study advances our understanding of indigenous psychology approaches to contextualizing religious faith. The findings have significant implications for mental health practitioners, policymakers, and community leaders who seek to leverage culturally embedded religious practices to enhance societal well-being.

## 6 Practical implications

This study highlights the significant role of religious belief in promoting mental health and fostering positive emotions. Based on these findings, it is recommended that religious groups and community organizations design targeted programs, such as workshops, support groups, and community events, aimed at alleviating stress, building resilience, and providing emotional support. These initiatives could effectively encourage greater public participation while addressing the mental health needs of individuals within the community. Moreover, organizations and businesses might consider integrating religious or spiritual practices into their employee wellness initiatives. Examples include mindfulness training, spiritual retreats, or providing access to spiritual counseling services. Such programs have the potential to not only enhance employees’ mental health and emotional well-being but also foster a greater sense of life satisfaction, thereby contributing to improved workplace productivity and overall quality of life.

## 7 Research limitations and research directions

Although the external validity of this study was enhanced by diversifying the sampling points and increasing the sample size, the use of convenience sampling may have introduced sample bias. Consequently, the findings should be cautiously generalized to all Mazu followers in Taiwan. Furthermore, a limitation of the questionnaire survey was the possibility that respondents might not have fully understood certain questions, which could potentially impact the accuracy of the data. These limitations need to be carefully considered when interpreting the results.

This research primarily focused on the belief in Mazu within the Taiwanese cultural context, and the findings may not be universally applicable. Future research could expand the scope of investigation to other regions or religions for cross-cultural comparisons. Additionally, the use of cross-sectional data in this study may limit the ability to establish causal relationships. Hence, longitudinal studies are recommended to explore the long-term effects of religious belief on mental health and overall well-being.

While this study employs a one-dimensional framework to simplify and emphasize the overall impact of Mazu belief, we acknowledge the potential for future research to adopt a multidimensional approach. It is therefore suggested that future studies could independently examine the effects of specific dimensions (e.g., belief practice versus belief ideology) on mental health and happiness, thereby deepening our understanding of the complex psychological mechanisms underlying Mazu belief.

## 8 Conclusion

This study examined the psychological effects of Mazu belief on the mental health, positive emotions, and happiness of Taiwanese followers, providing novel insights into the intersection of Eastern religious practices and psychological well-being. The findings demonstrate that Mazu belief significantly influences mental health and positive emotions while indirectly contributing to happiness through these two mediators. Although Mazu belief does not directly affect happiness, the identified mediating pathways underscore the capacity of religious faith to foster emotional resilience and psychological stability in culturally specific ways.

Compared to other Chinese and Asian religious traditions, such as Buddhism and Taoism, the results affirm both the shared and unique characteristics of Mazu belief in influencing emotional and mental well-being. For example, Buddhist traditions emphasize mindfulness and inner peace, while Mazu worship offers unparalleled communal and familial benefits steeped in Taiwanese traditions, including stress relief from familial obligations through ritualistic and intergenerational practices.

Theoretically, this study extends Fredrickson’s Broaden-and-Build Theory by showing that positive emotions generated through faith practices not only broaden cognitive capacities but also build enduring psychological resources. It also aligns with previous findings (Koenig et al., 2012; Ramsay et al., 2019) that highlight the mediating roles of mental health and positive emotions in explaining the indirect pathways from religion to happiness.

Moreover, the unique integration of collectivistic values within Mazu belief offers a contrast to the individual salvation focus of Western religions. The emphasis on relational harmony, family-oriented well-being, and community solidarity reflects religion’s role as an adaptive socio-cultural mechanism that reconciles individual psychological needs with broader collective obligations.

The findings of this study enrich our understanding of positive and cross-cultural religious psychology, shedding light on the substantial psychological benefits embedded within indigenous religious practices. Future research should explore inter-religious comparisons, longitudinal relationships, and the role of gendered archetypes, such as Mazu’s maternal symbolism, in influencing emotional well-being. By highlighting the psychological and cultural contributions of Mazu worship, this study underscores the enduring value of religious traditions in promoting mental, emotional, and communal wellness.

## Data Availability

The original contributions presented in the study are included in the article/supplementary material, further inquiries can be directed to the corresponding author.
